# Structural and Functional Analysis of Latex Clearing Protein (Lcp) Provides Insight into the Enzymatic Cleavage of Rubber

**DOI:** 10.1038/s41598-017-05268-2

**Published:** 2017-07-21

**Authors:** Lorena Ilcu, Wolf Röther, Jakob Birke, Anton Brausemann, Oliver Einsle, Dieter Jendrossek

**Affiliations:** 1 Institute for Biochemistry, Albert-Ludwigs-Universität Freiburg, Albertstrasse 21, 79104 Freiburg, Germany; 2 Institute of Microbiology, University of Stuttgart, Allmandring 31, 70550 Stuttgart, Germany; 3 BIOSS Centre for Biological Signalling Studies, Schänzlestrasse 1, 79104 Freiburg, Germany

## Abstract

Latex clearing proteins (Lcps) are rubber oxygenases that catalyse the extracellular cleavage of poly (*cis*-1,4-isoprene) by Gram-positive rubber degrading bacteria. Lcp of *Streptomyces* sp. K30 (Lcp_K30_) is a *b*-type cytochrome and acts as an *endo*-type dioxygenase producing C_20_ and higher oligo-isoprenoids that differ in the number of isoprene units but have the same terminal functions, CHO-CH_2_– and –CH_2_-COCH_3_. Our analysis of the Lcp_K30_ structure revealed a 3/3 globin fold with additional domains at the N- and C-termini and similarities to globin-coupled sensor proteins. The haem group of Lcp_K30_ is ligated to the polypeptide by a proximal histidine (His198) and by a lysine residue (Lys167) as the distal axial ligand. The comparison of Lcp_K30_ structures in a closed and in an open state as well as spectroscopic and biochemical analysis of wild type and Lcp_K30_ muteins provided insights into the action of the enzyme during catalysis.

## Introduction

Rubber, or caoutchouc, has unique physical properties and has been widely used by mankind for more than 100 years. As a result, our modern life is characterised by the ubiquitous presence of rubber-based products such as tyres, seals, latex gloves and many other items made from natural or synthetic rubbers. The main component of rubbery materials is the hydrocarbon poly (*cis*-1,4-isoprene), an ingredient of latex milk that is produced by a variety of plants and even by some fungi. The polymer is synthesized by specific *cis*-prenyltransferases from the C_5_-precursors isopentenyl diphosphate and dimethylallyl diphosphate^1^. The majority of currently used natural rubber materials is derived from the rubber tree *Hevea brasiliensis* that accumulates large amounts of rubber latex in specialized tissues, the lacticifers. While the cultivation of *H*. *brasiliensis* requires a tropical climate, other high-quality rubber-producing plants such as the Russian dandelion (*Taraxacum kok-saghyz*) thrive well in the northern hemisphere, even on nutrient-poor soils. This plant has thus become a model organism to investigate and to optimize rubber biosynthesis^2^.

Despite the economic importance of natural rubber and the considerable quantities of rubber waste that are permanently released into the environment, the knowledge about the fate of rubber materials in nature is still limited. Rubber-degrading microorganisms were isolated from various ecosystems and seem ubiquitous in habitats with moderate physical parameters (e.g. moderate temperature, pH etc.)^3–8^. For microorganisms, the use of rubber as a carbon and energy source depends on the ability to synthesize rubber-cleaving oxygenases and to transport these enzymes out of the cell to reach the insoluble polymeric substrate. The low molecular weight primary degradation products can then be taken up and metabolized by the cells via β-oxidation^9, 10^.

To date only two types of such extracellular rubber-cleaving enzymes have been reported. One is the rubber oxygenase RoxA that was first isolated from *Xanthomonas* sp. 35Y^11, 12^. Later, several RoxA homologs were described in other Gram-negative rubber degraders^13^. RoxAs are di-haem *c*-type cytochromes that cleave poly (*cis*-1,4-isoprene) into 12-oxo-4,8-dimethyl-trideca-4,8-diene-1-al (ODTD) as the predominant product in a dioxygenase reaction^14, 15^. The characterization of the active site of RoxA from *Xanthomonas* sp. (RoxA_Xsp_) by molecular biological^16^ and by structural analysis^17^ revealed that a dioxygen molecule is stably bound to the active site of RoxA_Xsp_ in the *as isolated* state. The second type of rubber oxygenase has been named latex clearing protein (Lcp)^18^. Since the first identification of an *lcp* gene in *Streptomyces* sp. K30 (*lcp*_K30_)^18^ many other *lcp*-like genes were described, for instance in rubber-degrading *Gordonia* species (*G*. *polyisoprenivorans*, *G*. *westfalica*)^6, 19, 20^, in *Rhodococcus rhodochrous* RPK1 (*lcp*_Rr_)^21^ and recently in other *Streptomyces* species^8^. Apparently Lcps are present in many other – if not all – Gram-positive rubber degraders^22^. So far, only three Lcps (from *G*. *polyisoporenivorans* VH2, *Streptomyces* sp. K30 and *R*. *rhodochrous* RPK1) have been purified and biochemically characterized^21, 23–26^.

The amino acid sequences of RoxA and Lcp largely differ in length and show no relevant similarity, but despite this dissimilarity both enzyme types have a monomeric quaternary structure and catalyse the oxidative cleavage of the double bonds in poly (*cis*-1,4-isoprene) and produce similar cleavage products with terminal keto and aldehyde groups. In contrast to RoxAs that cleave rubber in an *endo*-type, processive manner to a single major end product (ODTD, C_15_ oligoisoprenoid), Lcps produce a mixture of cleavage products that differ in the number of central isoprene units^24, 27^. Controversial reports were published on the cofactor of Lcp from *Streptomyces* sp. K30 (Lcp_K30_) and *G*. *polyisoprenivorans* VH2 (Lcp_VH2_)^23, 24^ but recently iron was detected in an almost 1:1 stoichiometry in pure preparations of Lcp_K30_ and of Lcp_Rr_, and both Lcps were identified as *b*-type cytochromes^21, 25^. Here we report the three-dimensional structure of Lcp_K30_ in a closed and in an open confirmation. Moreover, we provide insights into the function of the active site obtained by spectroscopic methods and by the evaluation of previously published and newly generated results from mutagenesis of amino acid residues close to the active site.

## Results and Discussion

### Lcp has a globin fold

Crystals of Lcp_K30_ belong to the triclinic space group *P*1 with two monomers (A and B) per asymmetric unit (residues 31-403, r.m.s.d. = 0.28 Å for 2119 atoms in the open state and 0.15 Å for 2282 atoms in the closed state, Table 1). Almost two thirds (63%) of Lcp_K30_ attain an α-helical secondary structure, with the remaining parts forming connecting loop regions. No β-strands were observed, and the absence of cysteine residues excludes the formation of disulphide bridges that were an unusual feature in the structure of the other rubber-degrading enzyme of known structure, RoxA^17^. Several internal salt bridges, including Asp56-Arg195 and Asp60-Arg202, or hydrogen bonds (His203-Glu68, Glu148-Thr230) stabilize the structure of Lcp. The importance of the strictly conserved residues Arg195 and Arg202 for protein stability was recently shown experimentally by site-directed mutagenesis^26^.

**Table 1:** Statistics of the data collection and structure refinement.

	Open state 5O1L	Closed state 5O1M
**PDB ID**		
resolution [Å]	60.37–1.48	39.22–2.20
	(1.51–1.48)	(2.27–2.20)
unit cell constants *a*, *b*, *c* [Å]	56.7, 62.8, 64.4	54.9, 56.6, 63.9
α, β, γ [°]	85.4, 66.1, 74.2	74.2, 86.1, 71.0
space group	*P* 1	*P* 1
unique reflections	117,382 (5,807)	30,914 (2,566)
multiplicity	3.6 (3.7)	3.7 (3.7)
completeness [%]	90.0 (89.2)	87.2 (83.7)
mean ((*I*)/σ (*I*))	8.1 (2.1)	12.3 (3.8)
CC (1/2)	0.994 (0.730)	0.997 (0.932)
*R*_merge_	0.086 (0.596)	0.065 (0.299)
*R*_pim_	0.053 (0.358)	0.039 (0.180)
**Refinement**		
*R*_cryst_	0.1649	0.1746
*R*_free_	0.1867	0.2253
r.m.s.d. bond lengths [Å]	0.0202	0.0184
r.m.s.d. bond angles [°]	2.0085	1.9487
non-hydrogen atoms	6,374	5,845
Ramachandran statistics		
*most favoured regions* [*n*, %]	732, 98.4	720, 97.6
*allowed regions* [*n*, %]	11, 1.5	18, 2.4
*outliers* [*n*, %]	1, 0.1	0

**Figure 1:**
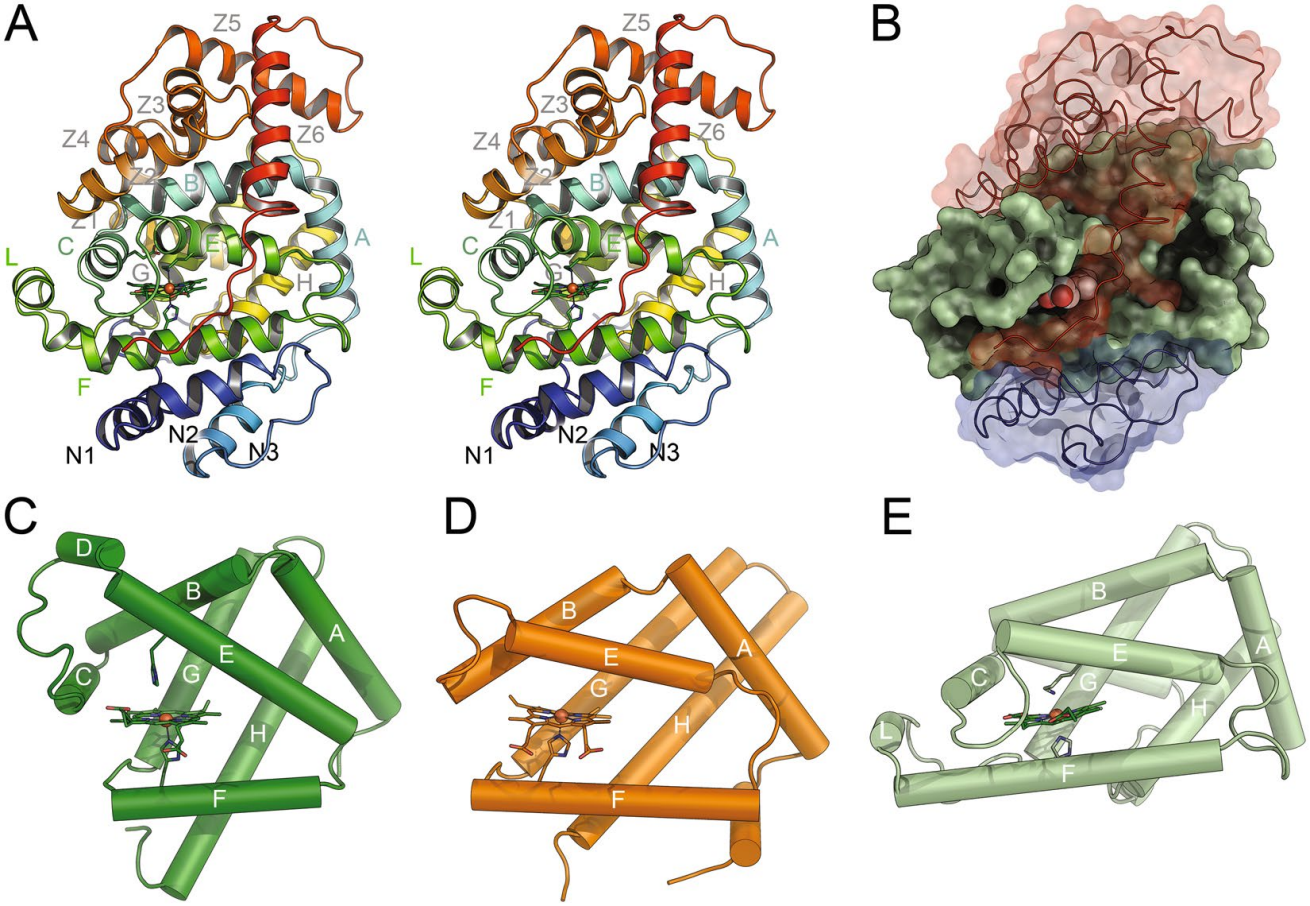
Lcp has a globin fold and similarities to globin-coupled sensor proteins. Three-dimensional structure of Lcp_K30_ (stereo image), coloured from blue at the N-terminus to red at the C-terminus (**A**). Helix designations follow the standard globin nomenclature. Note that Lcp_K30_ contains an additional helix, L, in the globin domain. Domain structure of Lcp_K30_ (**B**). A central domain with a globin fold (green) is capped by an N-terminal domain with three helices (N1-3, blue) and a C-terminal domain consisting of 6 helices (Z1-6, red). Secondary structures of sperm whale myoglobin (PDB-ID 1MBN) (**C**), the globin-coupled sensor protein of *G*. *sulfurreducens* (PDB-ID 2W31) (**D**) and the central domain of Lcp_K30_ (**E**) highlight the conserved topology of the globin folding core, as well as substantial variations in helix arrangement and haem accessibility. The haem groups including their axial ligands are shown as sticks in (**A**,**C**,**D**,**E**).

The typical, α-helical tertiary structure identifies Lcp_K30_ as a member of the globin family^28, 29^. A structural comparison to other globins revealed a close relationship of the protein core to myoglobin (PDB-ID 1MBN, r.m.s.d. = 3.69 Å) and to the globin-coupled sensor of *Geobacter sulfurreducens* (GCS_Gsu_, PDB-ID 2W31, r.m.s.d. = 2.97 Å) (Fig. 1)^30^. However, Lcp_K30_ contains additional subdomains at the N- and C-termini beyond the globin core that form two caps located on opposite sides of the protein. The N-terminal cap comprises three α-helices (N1–N3), while six of the secondary structure elements (Z1–Z6) form the C-terminal cap (Fig. 1). The central globin core attains the classical 3/3 globin fold with helices numbered A to H according to the common nomenclature for globins (Fig. 1A)^28^. Helix D is absent in Lcp_K30_, as is the case in GCS_Gsu_. A short α-helix, spanning residues Pro209 to Thr214, is additionally present between helices E and F. This helix includes a tryptophan residue (Trp211) that is generally conserved among Lcp sequences and was named the L-helix (for Lcp-specific helix, Fig. 1A,E). Its position prevents the free access to the haem cofactor from the surface of the molecule. The haem moiety itself is buried in an internal pocket of the Lcp globin core of helices A–H in its canonical location within the family. As a *b*-type cytochrome, the haem group is not covalently bound to the polypeptide, but fixed in place through a series of defined interactions (Suppl. Fig. S1).

### Unusual axial histidine-lysine coordination of the haem cofactor

His198 (F13) acts as the proximal axial ligand in Lcp_K30_ and corresponds to His (F8) of GCS_Gsu_ according to the haemoglobin nomenclature^31^. The importance of His198 for the binding and axial coordination of the haem cofactor was already reported^26^ and is confirmed by analysis of the structure of Lcp_K30_ in this study. The His198Ala Lcp_K30_ mutein could be expressed and purified as the wild type protein and no detectable change in CD-spectroscopy was observed. The His198Ala mutein was surprisingly stable but completely inactive indicating that the haem residue is not essential for a correct folding of the protein but pointed out that the haem cofactor is crucial to catalyse the oxidative cleavage of rubber.

Interestingly, distal haem ligation in Lcp_K30_ is not provided by a histidine as in most other globins and many cytochromes, but by a lysine residue (Lys167, E7) that corresponds to (His) E11 in GCS_Gsu_ and (His) E7 in haemoglobin. An axial ligation of a haem group by a lysine residue is rare in *b*-type cytochromes and was previously only described for the truncated haemoglobin THB1 of *Clamydomonas reinhardtii* ^32^ and for the putative globin-coupled sensor protein HGbRL of *Methylacidiphilum infernorum* ^33^. Interestingly, both proteins and Lcp_K30_ lack helix D. Structures of imidazole bound open and lysine ligated closed state structures of HGbRL show a large conformational change as observed for Lcp_K30_ (see below).

The presence of two axial ligands (His198, Lys167) to the haem iron in Lcp_K30_ prevents the direct binding of dioxygen that is required for the dioxygenase activity of the enzyme and identifies this structure as a closed and inactive form. As a consequence, a conformational change in the vicinity of the haem group is required to allow for access and binding of O_2_ and of the polyisoprene chain to the haem site. His198 and Lys167 are conserved in the other two biochemically characterized Lcps (Lcp_VH2_ and Lcp_Rr_)^23, 26^ suggesting that they also might constitute the axial haem ligands in these Lcps. However, while His198 is strictly (100%) conserved in an alignment of 495 Lcp sequences, the Lys167 residue is only moderately conserved (59%) in other Lcps and this might point to a variation of the distal axial coordination of haem in other Lcps.

### Conformational flexibility in Lcp_K30_

**Figure 2:**
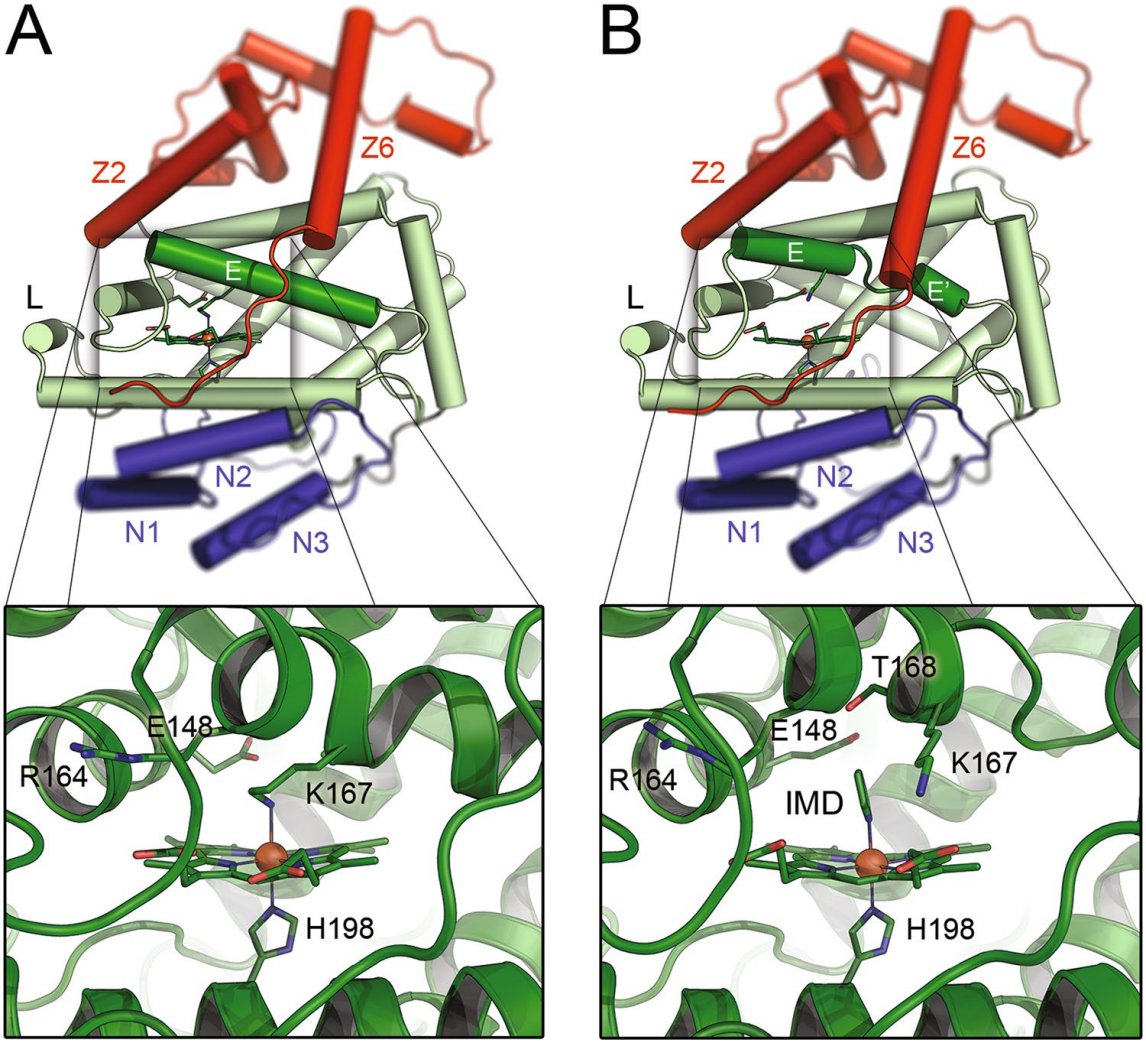
Two conformers of Lcp_K30._ The Lcp_K30_ structure exhibits an overall globin fold (**A**,**B**), and Lys167 serves as a distal axial ligand to the haem group, effectively preventing access for the substrate O_2_. The N-terminal extension with helices N1–N3 is shown in blue, the C-terminal with helices Z1–Z6 in red. In the presence of imidazole, an open form of Lcp_K30_ was obtained (**B**). Lys167 is removed as a distal axial haem ligand and helix E is split into two fragments. Imidazole binds tightly to the haem and a continuous substrate channel passing the haem group is opened (Fig. 3).

**Figure 3:**
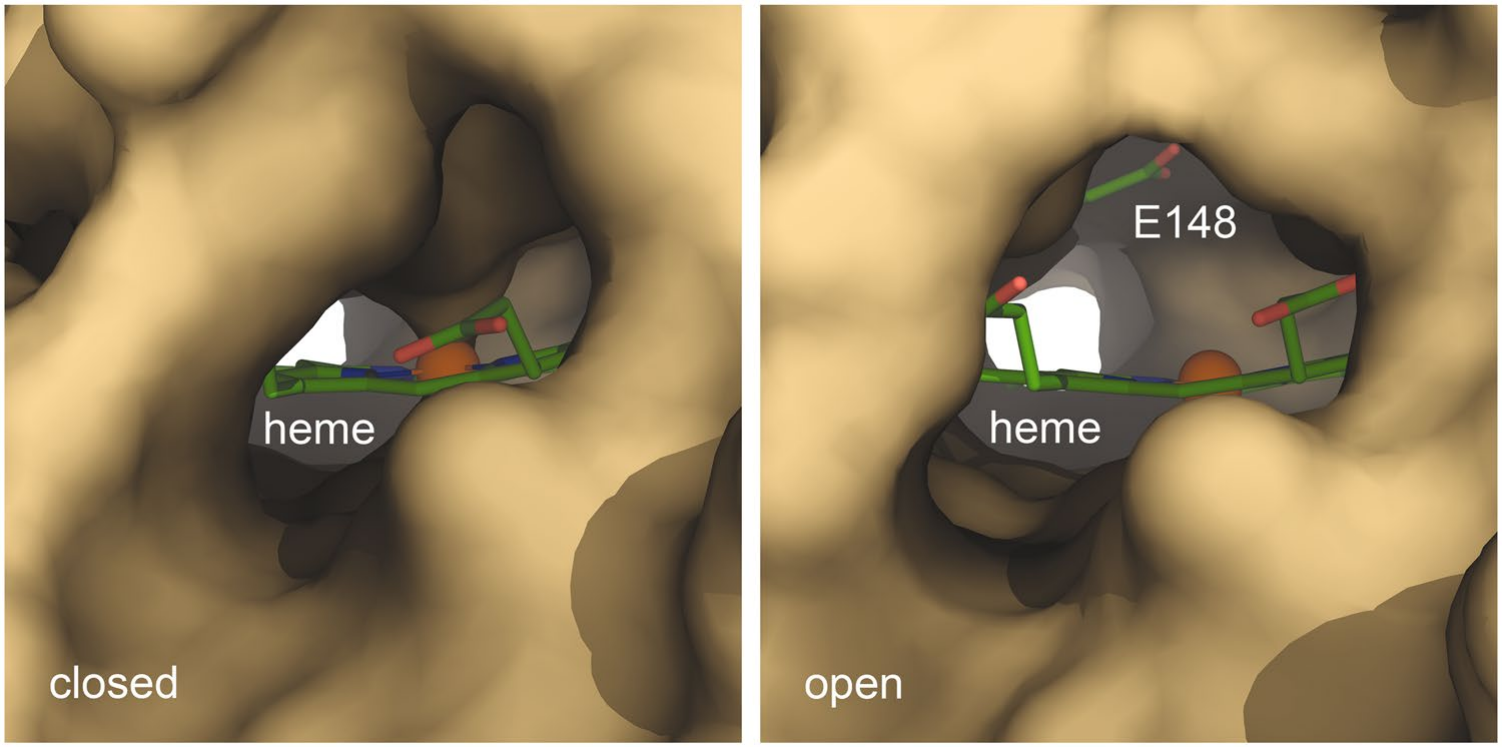
Comparison of the open and closed state of Lcp_K30_. A molecular surface view of the hydrophobic channel passing the active site haem shows that the ligation of the iron ion by residue Lys167 effectively blocks the entrance. The open state provides a continuous channel that is lined by hydrophobic residues only, with the notable exception of Glu148 that possibly acts as a base during catalysis. The hydrophobicity of the channel seems to prevent access for water, leaving the haem in a five-coordinate state prior to O_2_ binding.

While screening for appropriate crystallisation conditions, diffracting crystals of Lcp_K30_ were also obtained in an imidazole-containing buffer, and a structure of Lcp_K30_ in these crystals was determined to 1.48 Å resolution. These crystals belonged to the same space group (*P*1), albeit with slightly altered unit cell axes, again with two subunits per asymmetric unit. Most features of Lcp_K30_ remained unchanged in this structure, including the positioning of the proximal axial ligand, His198, towards the haem moiety (Fig. 2B). However, remarkable differences were found for the distal haem ligand Lys167 and for Thr168. Both residues were shifted significantly, releasing the coordinative bond to the metal ion and breaking the secondary structure of helix E into two shorter helices connected by a loop. Concomitantly, the C-terminal helix Z5 was split into two parts connected by a short loop, helix Z6 rearranged towards the active site and the distal axial position at the haem iron was now occupied by an imidazole molecule from the crystallisation buffer (Fig. 2B). This structure showed an increased accessibility of the haem group, opening a direct access pathway to a cavity at the distal side of the haem group (Fig. 3), and in spite of the presence of imidazole we suggest this to represent an open form of Lcp_K30_. The observation of two similar conformations that differ in the nature of the distal haem ligands confirms a flexibility of the Lcp structure at the distal haem site and suggests that Lcp is a member of the hexa-coordinate haemoglobin subfamily that can switch between a hexa- and penta-coordinated form^34^. The imidazole-bound Lcp could thus represent the structure of the otherwise penta-coordinated form that is able to bind dioxygen.

Spectroscopic data further confirmed this insight, since the addition of imidazole to dithionite-reduced Lcp_K30_ resulted in no observable increase of the absorption, whereas for Lcp of *R*. *rhodochrous* (Lcp_Rr_) a drastic increase was observable^21^. These different responses of two related Lcps to the addition of imidazole are thought to reflect a closed, 6-fold coordinated state in case of Lcp_K30_ *as isolated* in contrast to an open state with a 5-fold coordinated haem residue in case of Lcp_Rr_.

### Characterization of the Lcp_K30_ active site

We provide evidence that the distal haem site with Arg164 and Thr168 in the vicinity of the distal haem position Lys167 represents the active site of Lcp_K30_. The importance of both residues for Lcp activity was recently shown for purified Lcp_K30_ muteins^26^. The two Lcp_K30_ muteins Arg164Ala and Thr168Ala were stable and contained the haem cofactor, but showed only 2% residual activity compared to the wild type in case of Thr168Ala and no detectable activity for Arg164Ala. Both muteins were indistinguishable from the wild type with respect to their UV/vis spectroscopic properties in the *as isolated* state (see Fig. 3 of ref. 26). However, substantial differences were observed upon reduction with sodium dithionite. In particular, an increase of absorption at 430 nm and at 562 nm occurred upon the addition of imidazole to the reduced Thr168Ala mutein that was not observed for wild type Lcp_K30_. This indicated an open state in Thr168Ala, in comparison to a closed state of wild type Lcp_K30_.

To elucidate the ligation of the active site haem of Lcp_K30_, we exchanged the distal haem ligand Lys167 via site-directed mutagenesis of the *lcp* gene by an alanine residue that has a shorter and hydrophobic side chain. Since in most globins the haem group is ligated by two histidine residues, we also exchanged the unusual distal haem ligand Lys167 with a histidine residue (Lys167His). Both Lcp_K30_ variants were expressed in *E*. *coli* and purified from soluble cell extracts as described previously^26^. Both Lcp_K30_ muteins still harboured the haem cofactor (red colour of concentrated proteins) and were of high purity as revealed by SDS gel electrophoresis (Suppl. Fig. S2). They were still able to oxidatively cleave polyisoprene; however, the specific activities were reduced to 20% in case of Lys167Ala and to 12% for Lys167His while the product spectrum was unchanged (C_20_ and higher oligoisoprenoids, Suppl. Fig. S3). These results confirmed that the amino group of the lysine residue as an unusual haem ligand or the higher flexibility of a lysine side chain vs. a histidine is important for a full activity of the enzyme. Surprisingly, both Lys167 muteins showed distinctive spectroscopic details differing significantly from the wild type. The Lys167Ala mutein (Suppl. Fig. S4a,c,e) shows a blue-shifted Soret maximum at 407 nm as well as a 628 nm absorption band similar as it was observed for Lcp of *R*. *rhodochrous* (Lcp_Rr_), previously (Suppl. Fig. S4b)^21^. No increased absorption of the shifted Soret band (429 nm) and no defined Q-bands at 560 nm as well as the disappearance of the 628 nm band were observed after reduction with dithionite. When imidazole was added as a potential haem ligand to the reduced Lys167Ala mutein, a significant increase of absorption at 431 nm, and defined split Q-bands at 533 and 563 nm occurred. Addition of imidazole to the *as isolated* (oxidized) form of Lys167Ala (Suppl. Fig. S4e) resulted in a disappearance of absorbance in the 630 nm region and in a red-shifted Soret band to 414 nm. This behaviour correlates to the spectroscopic properties of myoglobin (see Fig. 1 of ref. 35) that shows a 5-fold coordination under oxidised conditions (metmyoglobin, Fe^3+^) characterised by a lack of defined Q-bands and strong absorption at ~630 nm. Reduced deoxymyoglobin on the other hand is characterised by a single Q-band (~560 nm) and shows a disappearance of the 630 nm band. After addition of imidazole, the Lys167Ala mutein shows spectroscopic properties that are characteristic for a 6-fold coordinated, reduced haem center^36^. These data suggest that the haem group (Fe^3+^) of Lys167Ala mutein is 5-fold coordinated and corresponds to the open form of Lcp_K30_. We conclude that the haem group of the Lys167His mutein is 6-fold coordinated by His167 and His198 and most likely represents a closed form of Lcp_K30_.

In summary, the findings for Lcp_K30_ muteins with single amino acid exchanges of residues in the active site region were in agreement with the 5-fold-coordinated (open) state of the Lys167Ala mutein and with the 6-fold coordinated (closed) state in the wild type and in the Lys167His mutein with His167 as a second axial haem ligand. These data confirm that the unusual Lys167 haem ligand stabilises the protein in the absence of substrate in the closed state. Furthermore, the unique properties of lysine are important for efficient catalysis that cannot be functionally replaced by histidine or alanine.

The comparison of the open and closed structure of Lcp_K30_ indicates a flexibility of the protein at the active site. We therefore postulate that residues Lys167 and Thr168 undergo a conformational change from the closed to an open state upon the binding of the substrates that grants access to the distal axial position of the haem group for substrate (dioxygen) binding. For a better visualization of the transition of Lcp_K30_ from a closed to an open state see the movie of Suppl. material video S6.

The transition of Lcp_K30_ to the open state generates a distinct, hydrophobic access channel that crosses the entire protein molecule leading past the active site haem group (Fig. 3) and exiting on the distal side. A single charged residue, Glu148, is located within this channel, at a distance of approximately 6 Å from the iron ion of the haem group. The transition to the closed state only bars one entrance to this tunnel, but concomitantly blocks access to the haem iron by reinstating Lys167 as a direct ligand to the metal, thus efficiently preventing the reaction of the enzyme with dioxygen. Although Lcp_K30_ is an extracellular enzyme, this conformational switch may well represent a protective mechanism to avoid the generation of reactive oxygen species from the reduced haem cofactor in the absence of substrate molecules. It thus might be the substrate itself whose insertion into the distal end of the substrate tunnel triggers the conversion to the open state. This is reminiscent of the situation in the well-characterized cytochrome P450 monooxygenases, where substrate binding sterically displaces a water molecule at the haem iron only then enabling the binding of dioxygen^37, 38^.

### Electron paramagnetic resonance (EPR) analysis confirms the presence of an open and closed state in Lcp_K30_

**Figure 4:**
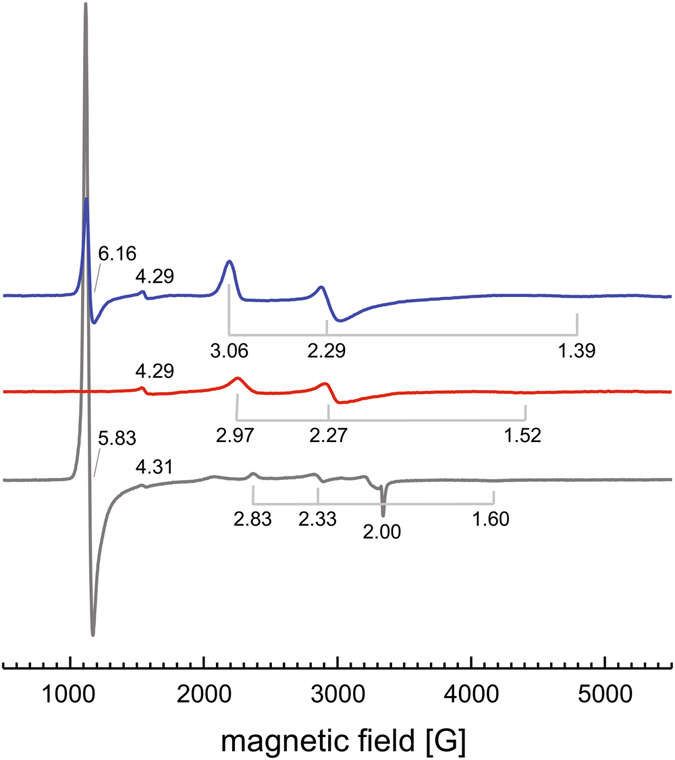
EPR analysis of Lcp_K30_. Continuous-wave EPR spectra were recorded for Lcp_K30_ *as isolated* (blue), Lcp_K30_ with imidazole (1 mM, red) and the Lcp_K30_ Lys167Ala variant (grey). Three discernible signals are a broad near-axial signal originating from a six-coordinate low-spin haem, most likely with Lys167 coordinating the haem iron. A signal at *g*~6 represents a second population of five-coordinate high-spin haem. Added imidazole binds to the haem group, replacing Lys167 and shifting the protein to a low-spin state. The high-spin signal disappears, and the *g*-values of the low-spin signal shift to 1.52, 2.27 and 2.97, representing the binding of imidazole, with the total population of paramagnetic Fe^3+^ being reduced.

Not uncommonly, continuous-wave X-band EPR spectra of the monohaem cytochrome *b* Lcp_K30_ *as isolated* showed at least two distinct paramagnetic species (Fig. 4, blue trace). A rhombic signal with resonances at *g*_x_ = 1.39, *g*_y_ = 2.29 and *g*_z_ = 3.06 is typical for a *low spin* haem (Fe^3+^, *S* = 1/2), presumably from the closed state of the enzyme with Lys167 completing the octahedral coordination of the metal ion. A second species with *g* = 6.16 then represents a *high spin* state (Fe^3+^, *S* = 5/2) that is frequently observed for five-coordinate haem and consequently originates from the open form of Lcp_K30_ that is present in an equilibrium with the closed state in the *as isolated* state in aqueous solution. A third, minor signal at *g* = 4.29 likely represents six-coordinate *high spin* Fe^3+^ as a non-specific residual in the sample. As crystals with the open conformation of Lcp_K30_ were only obtained in the presence of imidazole we also recorded the EPR spectrum in the presence of 1 mM imidazole (Fig. 4, red trace). Here the *high spin* signal at *g*~6 disappeared completely, indicating that the open state of the enzyme is capable of accepting imidazole as a distal ligand to haem, causing a transition to the *low spin* state. Interestingly, however, while still present the *low spin* signal slightly narrowed to *g*_x_ = 1.52, *g*_y_ = 2.27 and *g*_z_ = 2.97, completely replacing the previous resonances, so that this sample only shows a single haem species. This can be explained by imidazole not only binding to the open state of Lcp_K30_, but of also being able to displace the lysine ligand of the closed state, highlighting the intrinsic flexibility of the protein. In order to test the role of Lys167 in stabilizing the closed state of the enzyme we further investigated the properties of the Lys167Ala variant (see above) in the absence of imidazole. Its X-band EPR spectrum is strongly dominated by a *high spin* signal at *g*_||_ = 5.83 (Fig. 4, grey trace) that at this magnitude additionally shows an axial contribution at *g*_⊥_ = 2.00. This species represents the five-coordinate *high spin* Fe^3+^ haem that dominates in the protein in the absence of the distal lysine ligand, whereby the hydrophobic substrate channel (Fig. 3) prevents access of other ligands. That this exclusion is not complete can be seen from additional weak *low spin* signals with *g*_x_ = 1.60, *g*_y_ = 2.33 and *g*_z_ = 2.83. Again, this signal is narrower than both of the *low spin* species observed before and is thus likely distinct. Its low intensity points towards a hexacoordinate complex, possibly with the highly abundant H_2_O as a distal ligand. The presence of the Lcp_K30_ Lys167Ala mutein in the 5-fold coordinated (open) state is further confirmed by the strong increase of the Soret band intensity upon the addition of imidazole to the reduced protein (Suppl. Fig. S4).

All EPR spectra were recorded at the same protein concentration, making the overall signal intensities comparable. Most notably, the addition of imidazole not only led to the disappearance of the *S* = 5/2 *high spin* signal, but the intensity of the *low spin* signal also decreased markedly (Fig. 4). A loss of EPR intensity indicates that part of the haem groups attained a diamagnetic state that might be *low spin* Fe^2+^. Although at this point we cannot hypothesize on the origin of the electrons required for this reduction we note that only the Fe^2+^ state should be competent to bind O_2_ and is thus the catalytically relevant form.

### Carbon monoxide inhibits the polyisoprene cleavage reaction

**Figure 5:**
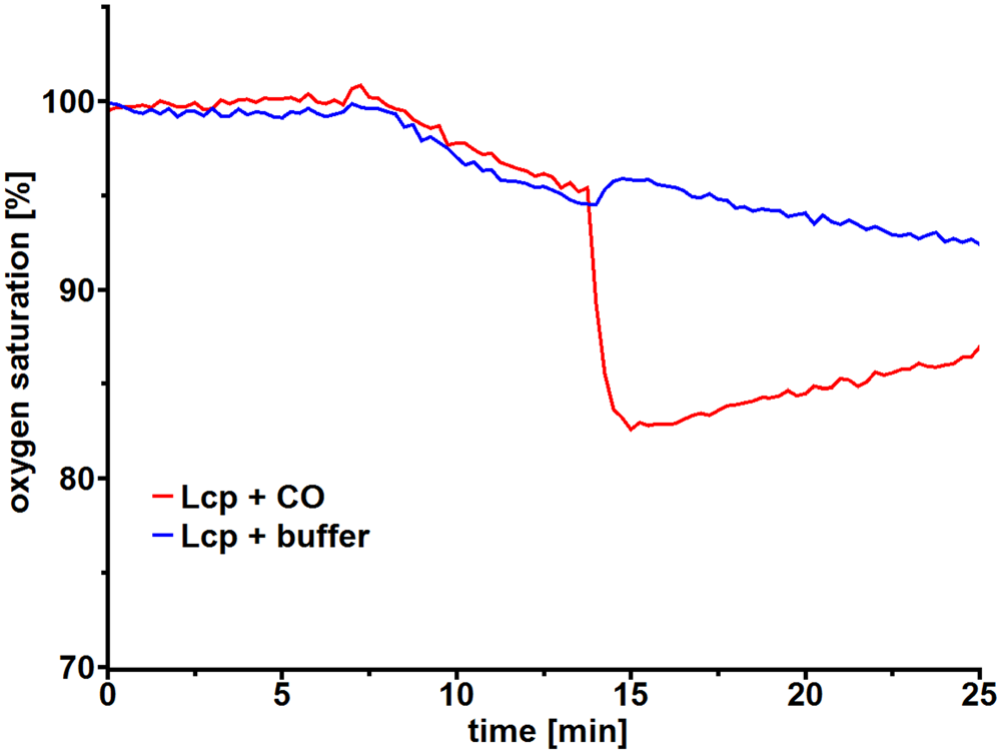
Inhibition of Lcp_K30_ by carbon monoxide (CO). The cleavage of a polyisoprene latex emulsion (1 ml) was initiated by the addition of 4 µg of purified Lcp_K30_ at t = 7 min (blue and red lines). The reaction was allowed to proceed until t = 14 min. At this time point, 200 µl of oxygen-saturated buffer (blue line) or 200 µl of a carbon monoxide-saturated buffer (red line) was added to the cuvettes. The dilution of the reaction buffer with the CO-buffer resulted in an immediate drop of the oxygen concentration. Note the continuous decrease of the oxygen consumption in the control (no CO, blue line). In contrast, the oxygen concentration did not decrease but increased in the CO-supplemented reaction (red line) due to diffusion from atmospheric oxygen.

EPR and UV/vis-spectroscopic analysis consistently showed that the haem iron of Lcp_K30_ *as isolated* is largely present in the ferric form, which has a low affinity for dioxygen. The addition of carbon monoxide to a solution of purified *as isolated* Lcp_K30_ did not change the UVvis spectrum in contrast to addition of CO to dithionite-reduced Lcp_K30_^21^. We assume that the Lcp-catalysed cleavage of polyisoprene at some point requires a reduction step of the Lcp-haem from the ferric to the ferrous form. To find further experimental evidence for an intermediate ferrous form of Lcp, we added carbon monoxide to an ongoing cleavage reaction of polyisoprene latex by purified wild type Lcp_K30_. To this end, Lcp_K30_ was added to a polyisoprene latex emulsion and the consumption of oxygen was recorded (Fig. 5). A constant decrease of the oxygen concentration was determined. However, when a carbon monoxide-saturated buffer solution was added to the ongoing reaction an immediate stop of the oxygen consumption was determined. This indicated that a carbon monoxide-sensitive, presumably short-lived reduced Lcp_K30_ species is present in the reaction cycle.

### Mechanistic considerations

**Figure 6:**
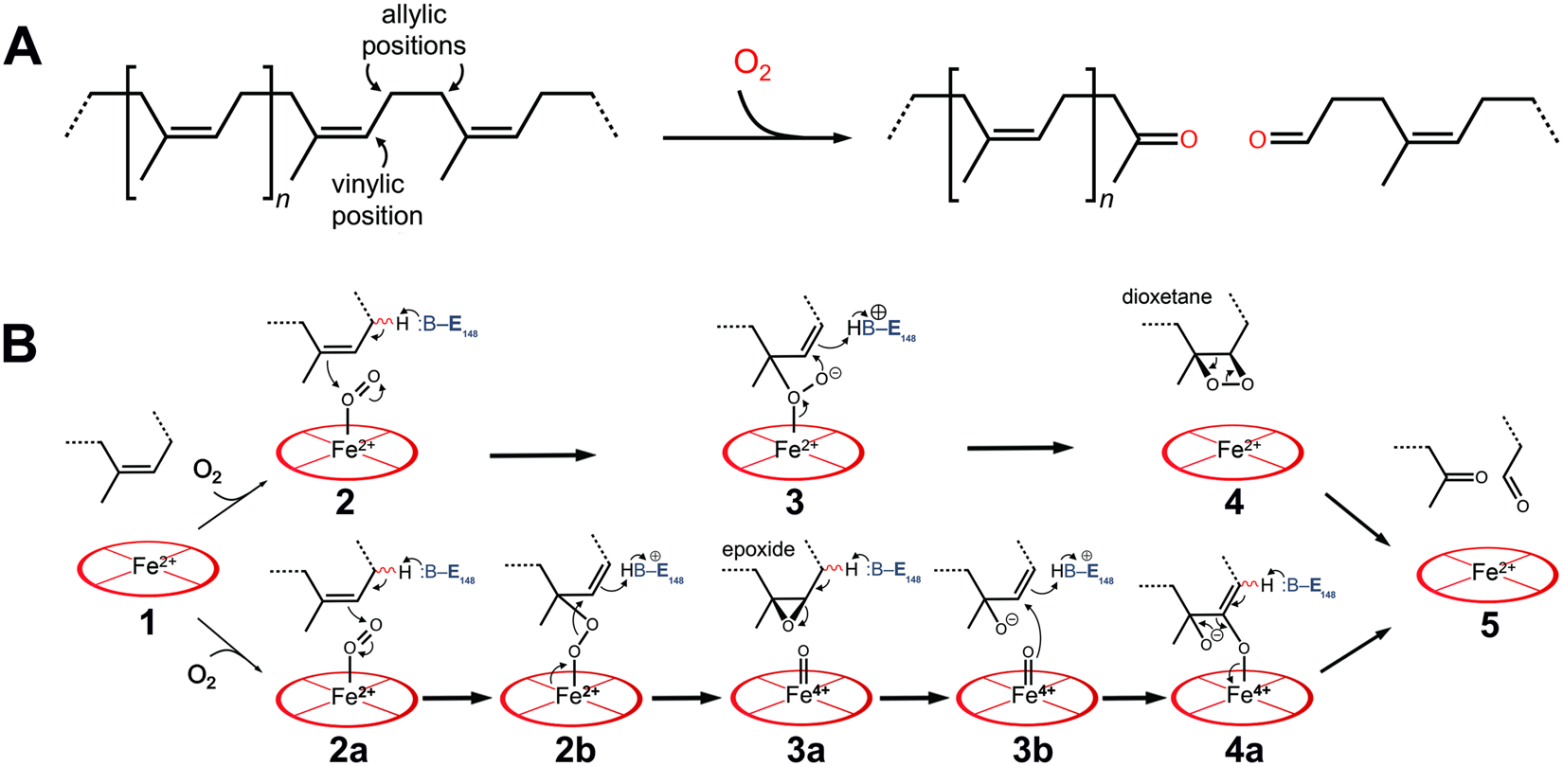
Mechanistic models of oxidative polyisoprene cleavage by Lcp_K30_. Protons in allylic positions of poly (*cis*-1,4-isoprene) will be more acidic than those in vinylic positions (**A**). LcpA_K30_ catalyses the cleavage of the isoprenoid by inserting both oxygen atoms of an O_2_ molecule. Possible reaction mechanisms (**B**). Top: The substrate polymer is threaded into the channel of Lcp_K30_ in the open state (1). O_2_ binds to the distal axial position of haem iron and a base in the channel, likely Glu148, abstracts a proton from an allylic position, leading to bond formation to an oxygen (2 + 2a). The second oxygen atom, with increased nucleophilic character, attacks the adjacent carbon (3), leading to the formation of an instable, cyclic dioxetane intermediate (4) that spontaneously rearranges to the cleaved product (5). Bottom: Alternatively, the haem-bound dioxygen can be cleaved (2b) to give a substrate epoxide and an oxo-ferryl intermediate (3a). The epoxide bond is cleaved by a nucleophilic attack of the oxo-ferryl-oxygen to the epoxide carbon atom (3b). Cleavage of the iron-oxygen bond (4a) leads to a release of the haem group and of the observed cleavage products (5).

Poly (*cis*-1,4-isoprene), the main constituent of natural rubber, is a chemically inert aliphatic polymer. Its activation and cleavage by molecular dioxygen presumably follows the mechanism discussed for haem-dependent dioxygenases such as tryptophan 2,3-dioxygenase (TDO) or indoleamine 2,3-dioxygenase (IDO)^39, 40^. In these enzymes, either a dioxygen molecule is bound to a ferrous haem or a superoxide molecule is bound to a ferric haem resulting in a heme-bound Fe^2+^−O_2_ species^41^. We have no direct evidence for the presence of such a haem-bound oxygen molecule in the *as isolated* form of Lcp_K30_ neither by UV/vis spectroscopy nor by structure determination of the present study. However, the strong inhibition of the Lcp-catalysed reaction by carbon monoxide (Fig. 5) indicates that Lcp_K30_ becomes reduced (ferrous) during the reaction cycle and in this reduced state the haem molecule can be readily oxygenated (Fig. 6). Conversion of the branched polymer is presumably initiated through proton abstraction, most likely by a base provided by the protein. The catalytically relevant state is the open form of the enzyme that features a hydrophobic channel crossing the entire molecule past the haem moiety, interrupted only by a single polar residue, Glu148. This glutamate residue is conserved in biochemically characterised Lcps. We assume that the polyisoprene chain is threaded through this channel, passing closely by Glu148 (Fig. 3). Each isoprene monomer contains one vinylic and four allylic hydrogen positions, of which the latter will show substantially elevated acidity and are thus the likely candidates for proton abstraction by Glu148. This state allows for different reaction pathways that are currently discussed in literature for TDO and IDO^37–41^. Neither pathway can be excluded for Lcp at present, therefore this contribution proposes two possible alternatives (Fig. 6B). Deprotonation of the substrate by Glu148 will allow for bond formation of one carbon atom of the isoprene double bond to one atom of the dioxygen molecule. The other oxygen atom can now perform a nucleophilic attack of the other carbon atom of the former carbon-carbon double bond to yield a cyclic dioxetane intermediate (Fig. 6B upper pathway). This will lead to the spontaneous cleavage of the isoprene polymer into the observed keto and aldehyde products^24^. Alternatively, as discussed in literature^40, 42, 43^, the haem-bound two oxygen atoms can be also consecutively inserted into the substrate by formation of a substrate epoxide and an oxo-ferryl-intermediate (Fig. 6B lower pathway). The oxo-ferryl intermediate can then transfer the oxygen atom to the epoxide releasing the observed polyisoprene degradation products and restoring the initial state of the enzyme.

### Glu148 is important for rubber oxygenase activity

To find experimental support for our assumption that Glu148 is important for the polyisoprene cleavage reaction we exchanged the codon for Glu148 against alanine, histidine and glutamine codons and purified the respective Lcp_K30_ Glu148 muteins. No significant differences in the biochemical and biophysical properties (Suppl. Fig. S4f–h) were detected during expression and purification of the Lcp muteins in comparison to the wild type protein. UV/vis spectra of the three purified muteins showed a wild type typical spectrum of the closed form of Lcp_K30_ and no substantial difference to the wild type could be detected. However, the specific activity of the Glu148Ala mutein was substantially reduced and only ≈20% of residual activity was determined. The specific activities of the Glu148Gln and the Glu148His muteins were even stronger reduced to 5% and 2% of the wild type value. The oligoisoprenoid product spectra of the cleavage reactions by all Glu148 Lcp_K30_ muteins were not changed (Suppl. Fig. S3). These results are in agreement with a prominent although not essential function of the glutamate residue at position 148.

Note that contrary to the situation in RoxA, where ODTD is the main product, Lcp_K30_ produces a diverse spectrum of isoprenoid oligomers. This indicates that the substantially smaller Lcp_K30_ protein (compared to RoxA) lacks the extent to generate a binding site for the end of the polyisoprene chain, a suspected basis for an internal ‘molecular ruler’ mechanism that assures a homogeneous product spectrum, which in turn is helpful to supply dedicated uptake systems in the bacterial outer cell wall or membrane. Lcp_K30_ may thus represent a more basic type of rubber dioxygenase as compared to RoxA.

## Conclusions

Overall, our findings contribute to illuminating the enzymatic mechanism of the oxidative rubber cleavage by Lcp and correlate the structure of Lcp_K30_ solved in this study with previous and newly obtained molecular insights. Lcp was classified as a globin and the structural importance of several residues contributing to the stability of the protein, especially Arg195 and Arg202 was stated. The conserved residues (Arg164, Thr168 and His198) of the recently biochemically characterised Lcp-specific domain of unknown function 2236 (DUF2236) are located close to the haem cofactor and were identified as crucial active site residues. The elucidation of open and closed state structures supports the postulation of a conformational change in the protein structure during the oxidative cleavage of polyisoprene, depending on Lys167 as an unusual distal haem ligand. The function of the charged Glu148 residue in an otherwise hydrophobic substrate tunnel as a base that facilitates the cleavage reaction was shown by drastically reduced activities of Lcp_K30_ muteins with substitutions at position 148.

## Methods

### Purification of Lcp_K30_ and of Lcp_K30_ muteins

In this contribution all protein variants with (single) amino acid exchanges obtained via mutation of the respective gene are designated as muteins. Wild type Lcp_K30_ and Lcp_K30_ muteins were isolated from the combined culture fluids of 8 individual 600 ml *Escherichia coli* JM109 cultures harbouring p4782.1::*lcp*_K30_ by two subsequent chromatographic steps (affinity chromatography on a StrepTactin resin (IBA Lifesciences, Göttingen) and size exclusion chromatography, Sephadex 200) exactly as described in detail recently^25^. All steps were performed under oxic conditions (normal atmosphere) and with oxic buffer solutions if not stated otherwise. The isolated proteins without any additions are referred to as “*as isolated*” in this study. Solutions of purified Lcp_K30_ proteins that have been treated with an excess of sodium dithionite are referred to as chemically reduced proteins. The Lcp-containing fractions were pooled, concentrated by ultrafiltration (10 kDa molecular weight cut-off) and stored on ice for up to one week. Alternatively, Lcp_K30_ was frozen in liquid nitrogen and kept in liquid nitrogen or stored at −70 °C for long term storage.

### Crystallisation and data collection

Lcp_K30_ was crystallised by sitting-drop vapour diffusion. 0.3 µl of protein solution (10 mg·ml^−1^) were mixed with 0.3 µl of precipitant solution using an OryxNano liquid dispensing system (Douglas Instruments) and equilibrated against the same reservoir solution at 293 K. The open form of Lcp_K30_ was crystallised with a precipitant solution containing 4% (*w*/*v*) of polyethylene glycol 4000 and 0.2 M malate/imidazole buffer at pH 7.5, while the closed conformation of the protein was obtained with 16% (*w*/*v*) of polyethylene glycol 3350, 0.2 M L-proline and 0.1 M HEPES/NaOH buffer at pH 8.5. For cryoprotection, single crystals were transferred through a reservoir buffer that additionally contained 10% (*v*/*v*) of 2*R*-3*R*-butane diol, mounted in nylon loops and flash-cooled in liquid nitrogen. Diffraction data were collected at 100 K on beam lines X06SA and X06DA of the Swiss Light Source (Villigen, CH), using Pilatus 6 M and Pilatus 2 M pixel detectors (Dectris), respectively. For phase determination by single-wavelength anomalous dispersion (SAD), a high-multiplicity data set was collected at the Fe K-edge at a wavelength of 1.73 Å, combined from three individual passes at different χ-angles (0°, 5°, 10°) of the PRIGo multi-axis goniometer^44^. Diffraction data were indexed and integrated with XDS^45^ and scaled and merged with AIMLESS^46^.

### Structure solution and refinement

Phasing, density modification and initial model building were carried out in AutoSol from the PHENIX suite^47^. The initial model obtained from automated model building was completed and corrected in COOT^48^ with iterative cycles of refinement in REFMAC5^49^. The final model for the SAD-phased data set was then used to phase a high-resolution data set at 1.4 Å resolution using PHASER^50^ and refined as described above. Structures were validated using MOLPROBITY^51^, and images were generated in PyMOL^52^. PDB coordinate files for LcpK30 were submitted to the Protein Database in Europe (PDBe) and are accessible under the identifiers 5O1L for the open state and 5O1M for the closed state.

### Site-directed mutagenesis of the *lcp*_K30_ gene

Site-directed mutagenesis of the *lcp*_K30_ gene and subsequent cloning and expression of the mutant *lcp*_K30_ gene was performed by the QuikChange method as described in detail previously^26^ using oligonucleotides shown in Suppl. Fig. S5.

### Lcp activity assay and determination of cleavage products

The activity of Lcp_K30_ was determined by the fluorescence-based online measurement of the oxygen concentration using an OXY-4 mini apparatus (PreSens, Regensburg, Germany) as described previously^24, 25, 53^. Poly (*cis*-1,4-isoprene) latex was diluted with 100 mM potassium phosphate buffer (pH 7) to 0.2% in an assay volume of 0.5 ml and incubated in the presence of purified Lcp_K30_ protein at 22 °C. Subsequently, cleavage products were extracted with ethyl acetate, evaporated, dissolved in methanol and applied to an RP8 HPLC column. Separation was achieved by an increasing methanol:water gradient as described earlier^24, 25, 53^.

### EPR spectroscopy

Perpendicular-mode X-band EPR spectra were recorded on a Bruker Elexsys E500 instrument with a 10″ ER073 electromagnet and a Super High Q resonator cavity. The system was equipped with an Oxford Instruments ER 41112HV continuous flow liquid helium cryostat controlled by an ITC 503 temperature device. The measurements were carried out at a temperature of 10 K and a power of 10 mW at ~9.38 GHz, with a modulation amplitude of 6 G and a receiver gain of 60 dB. A sample volume of 250 µl was used in 4 mm quartz tubes (705-PQ-9.50, Wilmad).

### Other techniques

Protein concentrations were determined by the bicinchoninic acid (BCA) method^54^. Concentrations of purified Lcp_K30_ samples were determined by molar extinction coefficients of Lcp at 412 nm (ε_412_ = 80,000 M^−1^ cm^−1^). Electron excitation spectroscopy was conducted as described previously^25^.

## Electronic supplementary material

Flexibility of LcpK30

Suppl material
