# Supplementary material for: Structural and Functional Analysis of Latex Clearing Protein (Lcp) Provides Insight into the Enzymatic Cleavage of Rubber
Source: Sci Rep. 2017 Jul 21;7:6179. doi: 10.1038/s41598-017-05268-2 (PMC5522427; doi:10.1038/s41598-017-05268-2)
Supplement: Supplementary file 2 — Suppl material [file 41598_2017_5268_MOESM2_ESM.pdf]

Structural and Functional Analysis of Latex Clearing Protein (Lcp) Provides Insight into the Enzymatic Cleavage of Rubber

Supplementary materials

Lorena Ilcu,[1,] Wolf Röther,[2,*] Jakob Birke,[2] Anton Brausemann,[1]*
Oliver Einsle,[1,3,†] Dieter Jendrossek[2,†]

1 Institute for Biochemistry, Albert-Ludwigs-Universität Freiburg, Albertstrasse 21, 79104 Freiburg, Germany

2 Institute of Microbiology, University of Stuttgart, Allmandring 31, 70569 Stuttgart, Germany

3 BIOSS Centre for Biological Signalling Studies, Schänzlestrasse 1, 79104 Freiburg, Germany

* these authors contributed equally

† Corresponding authors.

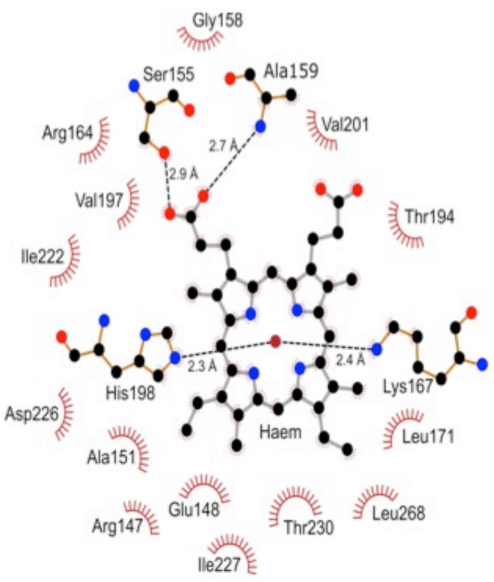

Suppl. Fig. S1: Ligation-plot of the haem cofactor of $\mathbf{Lcp_{K30}}$. As a *b*-type cytochrome, the haem group is not covalently bound to the polypeptide, but fixed in place through a series of defined interactions, as illustrated. The haem moiety is buried in an internal pocket of the Lcp globin core of helices A-H in its canonical location within the family. The plots on the left and right refer to the closed and open structure of Lcp_{K30}, respectively. The distances of the axial haem ligands to the haem iron are indicated.

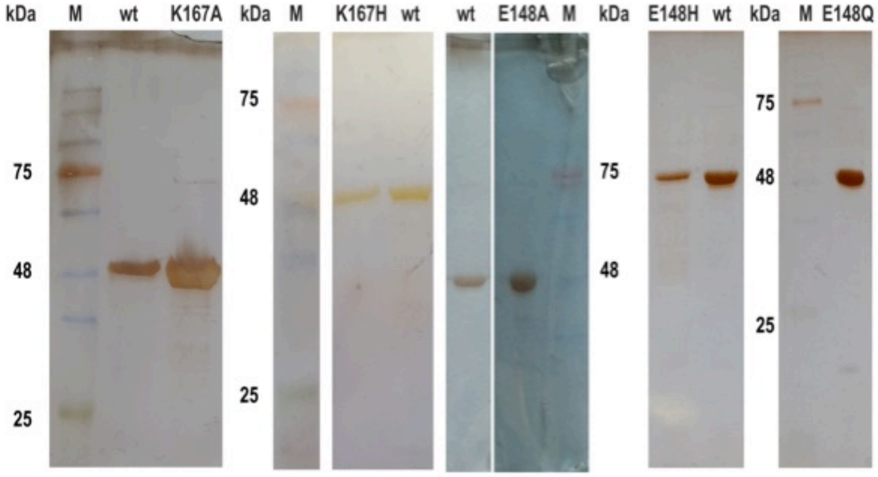

Suppl. Fig. S2: SDS-PAGE of purified Lcp$_{K30}$ muteins. The purity of isolated wild type (wt) Lcp$_{K30}$ and of Lcp$_{K30}$ muteins with exchanged residue as indicated was determined by SDS-PAGE and subsequent silver-staining. (M) refers to pre-stained molecular mass standards.

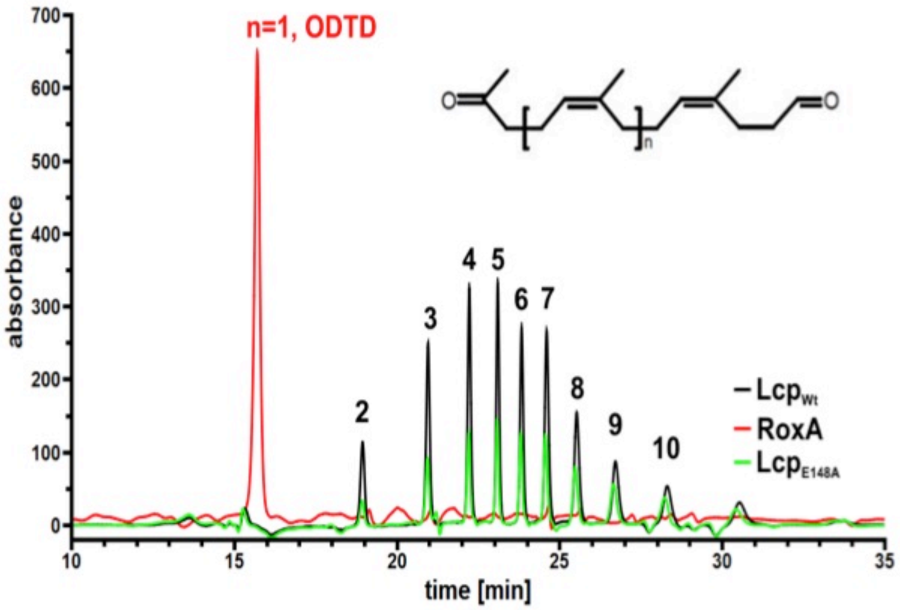

Suppl. Fig. S3: Poly(*cis*-1,4-isoprene) products of rubber oxygenase catalysed cleavage reaction. Polyisoprene latex was allowed to react with a purified rubber oxygenase as indicated (RoxA, Lcp_{K30}, Lcp_{K30} muteins) for 2 h. The produced cleavage products were solvent-extracted and separated by HPLC as described elsewhere [1]. RoxA of *Xanthomonas* sp. 35Y was included for comparison and produced the C_{15} compound ODTD as the only end product. The product spectra of all Lcp_{K30} proteins (wild type and all Lcp_{K30} muteins of this study) were qualitatively identical and differed only in the amounts of produced oligoisoprenoids as stated in the main text.

Suppl. Figs. S4: UV/vis spectra of Lcp$_{K30}$ wild type and of Lcp$_{K30}$ muteins purified in this study. Black lines always refer to the *as isolated* states, red lines to the states after reduction with sodium dithionite and green lines to the spectra that were recorded after the addition of 10 mM imidazole to the reduced protein and incubation for 10 to 15 minutes at room temperature. The Q-band region is enlarged in the insets.

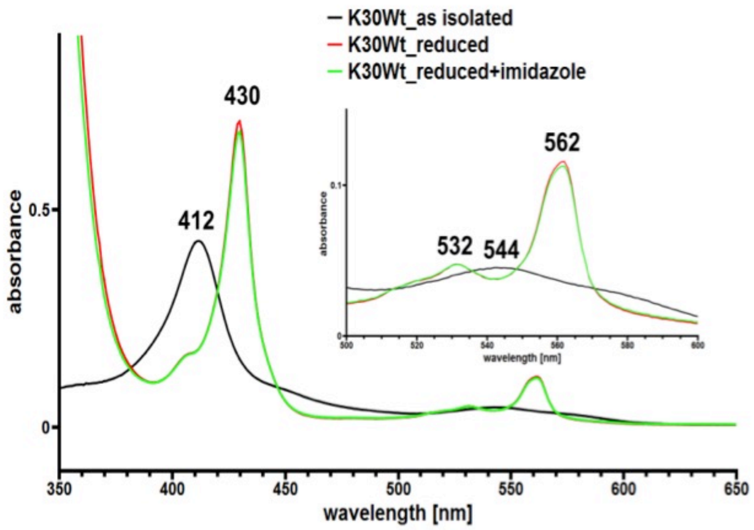

a. **Lcp$_{K30}$ wild type protein.** Note, no increase of Soret band intensity at 430 nm upon addition of imidazole to the reduced protein, representing the spectrum of the 6-fold coordinated heme in the *as isolated* state.

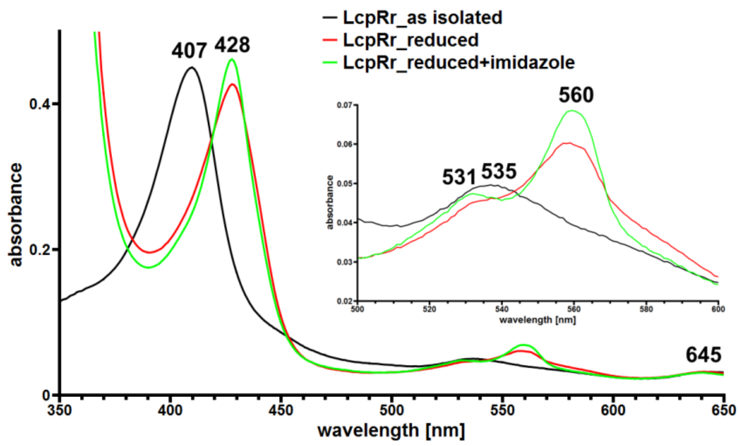

b. Lcp$_{Rr}$ wild type. In the *as isolated* state (black) the Soret band shows a maximum at 407 nm as well as a 535 nm band. After chemical reduction (red line) a shift to 428 nm occurs and the Q-bands have maxima at 531 and 560 nm. A strong increase of absorbance at 428 and 560 nm after addition of imidazole (green line) is observable, indicating an open, accessible state suggesting the absence of a second axial heme ligand.

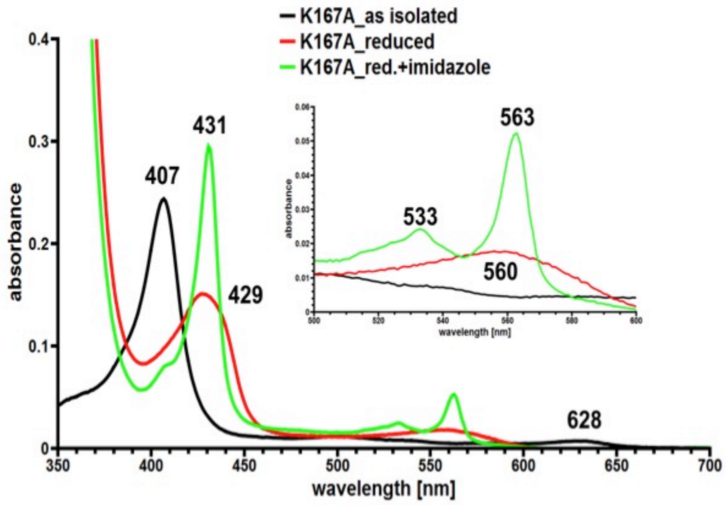

c. Lcp$_{K30}$ Lys167Ala mutein. Note the lack of defined q-bands after reduction and a strong increase of Soret band intensity at 431 nm upon addition of imidazole to the reduced protein as well as an absorbtion band at 628 nm in the *as isolated* state of the mutein, indicating a 5-fold coordinated form of Lcp$_{K30}$.

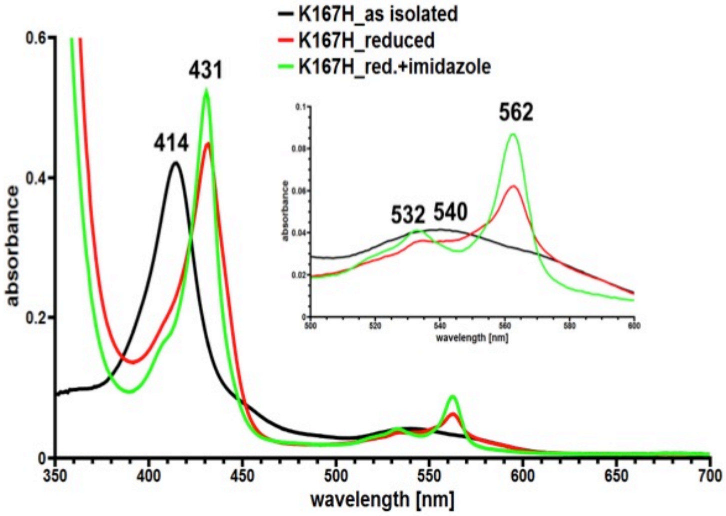

d. Lcp$_{K30}$ Lys167His mutein. Note moderate increase of Soret band intensity at 431 nm upon addition of imidazole to the reduced protein.

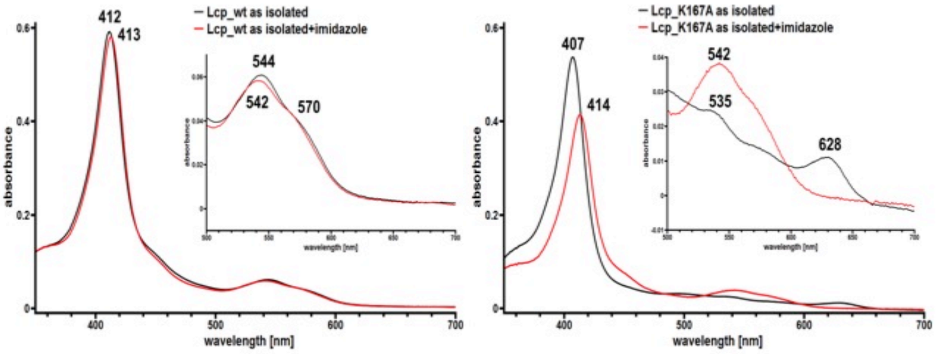

e. **Lcp$_{K30}$ wild type and Lcp$_{K30}$-Lys167Ala after addition of imidazole to the proteins in the *as isolated* states.** Note the Soret band at 407 nm observed for the mutein and a 628 nm absorption band comparable to the open state of Lcp$_{Rr}$ in the *as isolated* form [2]. A shift of the Soret band is noted and the 628 nm band disappears in the presence of imidazole indicating a now six-fold coordinated haem corresponding to the Lcp$_{K30}$ wild-type spectrum.

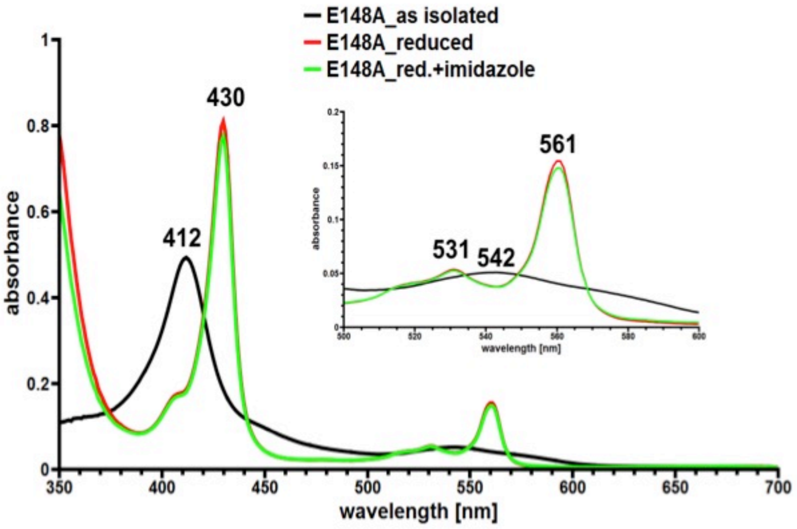

f. **Lcp$_{K30}$ Glu148Ala mutein.** Note no increase of Soret band intensity at 430 nm upon addition of imidazole to the reduced protein as it was observed for the wild type protein

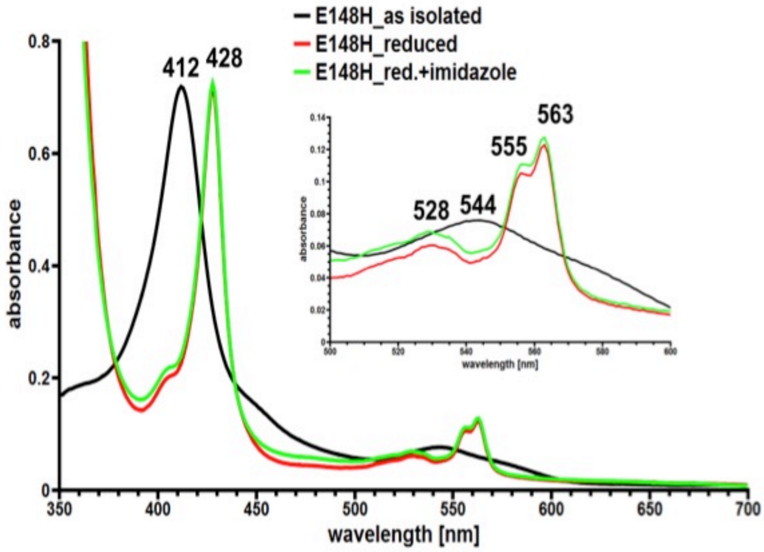

g. Lcp$_{K30}$ Glu148His mutein. Note no increase of Soret band intensity at 428 nm upon addition of imidazole to the reduced protein. A split alpha-band with maxima at 555 nm and 563 nm occurs, possibly triggered by distortion of the planar symmetry of the haem molecule. [3]

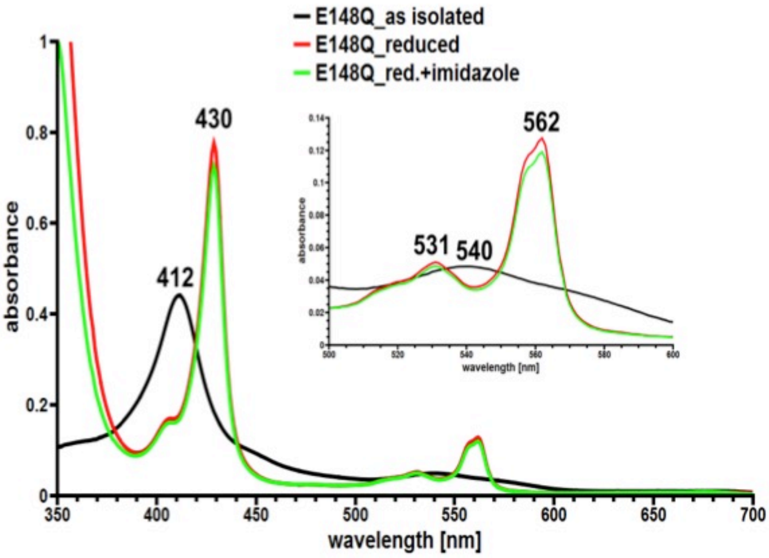

h. Lcp$_{K30}$ Glu148Gln mutein. Note, no increase of Soret band intensity at 428 nm upon addition of imidazole to the reduced protein

Suppl. Fig. S5: **Oligonucleotides used for mutagenesis for *lcp*$_{K30}$ muteins**

K167A	ACCGAATCGCTGCGACCGCACGTCTCGGT
RK167A	A ACCGAGACGTGCGGTCGCAGCGATTCGGT
K167H	ACCGAATCGCTCATACCGCACGTCTCGGT
RK167H	ACCGAGACGTGCGGTATGAGCGATTCGGT
E148A	TGCCATACCCAGGGCGTCGCGCGCCGTGT
RE148A	AACACGGCGCGCGACGCCCTGGGTATGGCA
E148H	TGCCATACCCAGGCACTCGCGCGCCGTGT
RE148H	ACACGGCGCGCGAGTGCCTGGGTATGGCA
E148Q	TGCCATACCCAGGCAGTCGCGCGCCGTGT
RE148Q	ACACGGCGCGCGACTGCCTGGGTATGGCA

Suppl. material movie S6: PyMol animation of the transition between the open and closed conformations of Lcp_{K30}. Important residues are represented by sticks (E148, R164, K167 and H198). See separate file.

References

1. Birke, J., Röther, W. & Jendrossek, D. Latex Clearing Protein (Lcp) of *Streptomyces* sp. Strain K30 Is a *b*-Type Cytochrome and Differs from Rubber Oxygenase A (RoxA) in Its Biophysical Properties. *Appl. Environ. Microbiol.* **81,** 3793–3799 (2015).
2. Watcharakul, S. *et al.* Biochemical and spectroscopic characterization of purified Latex Clearing Protein (Lcp) from newly isolated rubber degrading *Rhodococcus rhodochrous* strain RPK1 reveals novel properties of Lcp. *BMC Microbiol.* **16,** 92 (2016).
3. Srivastava, T. S. A carbon monoxide derivative of ruthenium (II) myoglobin probe of heme protein conformation. *Biochimica et Biophysica Acta (BBA) - Protein Structure* **491,** 599–604 (1977).
